# Supplementary material for: pyDHM: A Python library for applications in digital holographic microscopy
Source: PLoS One. 2022 Oct 10;17(10):e0275818. doi: 10.1371/journal.pone.0275818 (PMC9551626; doi:10.1371/journal.pone.0275818)
Supplement: Attachment — Submitted filename: Response_To_Reviewer_FINAL.pdf [file pone.0275818.s001.pdf]

Detailed response to reviewer's comments for PLOS ONE manuscript (manuscript ID: PONE-D-22-18805)

The authors thank the anonymous reviewer for reading the manuscript and for his/her effort in providing constructive criticism and positive feedback. A detailed reply to the reviewer's comments is provided below. Our responses are written in blue font below each reviewer's comment, and the corresponding manuscript changes are provided in red font for clarity. We have numbered the reviewer's comments. In cases where numbers were not provided, we have numbered the key points asked so that each point could be addressed clearly.

Reviewer 1

Comment #0: "The manuscript is clearly written. The authors conducted a thorough literature review in the field of digital holographic microscopy. Discussions on a broad range of DHM optical configurations and reconstruction methods are supported by appropriate references. The key innovation in this work is the proposal of the pyDHM library as a complete toolbox for numerical processing of holograms recorded from DHM systems of different configurations (on/slightly-off/off axis) with varying optical (telecentricity, wavelength, sensor properties) and acquisition parameters (focusing, phase-shifting intervals). However, there are several inconsistencies between the manuscript and the pyDHM library that require immediate corrections."
Response: We appreciate the kind words from the reviewer. The revised manuscript has been modified to address the multiple inconsistencies between the manuscript and the pyDHM library.

Comment #1: "Nevertheless, the pyDHM library provides a simplified approach in generalizing DHM numerical processing, which is beneficial for the field. I believe that the manuscript can be published in PLOS ONE as a research article, if the authors address the following comments."
Response: We hope to have adequately addressed the reviewer's comments so that the revised manuscript can be published in PLOS ONE as a research article.

Comment #2: "As a coding/programming-orientated work, a thorough documentation for the pyDHM library should be composed and included on the GitHub repository for this work."
Response: We have included complete documentation for the pyDHM library on the GitHub repository. Please refer to the revised GitHub repository: https://catrujilla.github.io/pyDHM/.

Comment #3: "The manuscript demonstrates sample codes for numerical processing, however, is not sufficient for facilitating troubleshooting. A detailed documentation that is standalone can immediately help readers and users trail the sample data, adopt this work to their workflow, troubleshoot, and contribute."
Response: The revised GitHub repository has been updated to become standalone. We have created four YouTube videos [R1-R4] to show users how to install and use the library, aiming for fast adoption of the pyDHM library in their research.

[R1] https://youtu.be/h76nZM6JpXo
[R2] https://youtu.be/Z9o0ODe1lUQ
[R3] https://youtu.be/CMHbF0uoWDk
[R4] https://youtu.be/3p6Bsh048Hw

Comment #4: "The pyDHM library should also incorporate appropriate error messages to guide the users upon error."
Response: Several error messages have been incorporated into the library to guide the users upon errors during its use to address the reviewer's comment. In particular, we have created error messages for five particular situations. The first two situations are related to the running environment and having the correct

libraries installed. In particular, error messages are displayed if the OpenCV (*cv2*) and *scipy* libraries are not installed. An error message is displayed when users incorrectly use one of the functions based on the given holograms. In other words, we have implemented a local function, named *regime*, within the in-line PS techniques (e.g., PS3, PS4, and PS5) and the telecentric-based phase compensation methods (e.g., FRS, ERS, and CFS). The *regime* function checks the optical configuration of the DHM system (e.g., on/slight off axis versus off axis) based on the spectrum of the input hologram and determines if the called function is suitable for the given hologram. For example, let us assume that the three terms in the hologram's spectrum overlap (e.g., non off-axis configuration) and the user calls the ERS function. Since that function is only valid for holograms recorded in off-axis configuration, an error message (e.g., 'ERS equires an off-axis hologram') is displayed. We have also implemented error messages when incorrect values of *s* and *step* parameters are used for the SORS, FRS, and ERS functions. Finally, we have included a new option into the *CNT* function (e.g., the phase compensation function for non-telecentric holograms). This option allows the user to decide how to perform the spatial filtering of the object frequencies from the hologram spectrum (please refer to the following response). An error message will appear if the user tries to call the *CNT* function using the x1, x2, y1, y2 parameters with the new option '*sfmr*'. Also, another error message will appear if the user cakks the CNT function using the '*sfr*' option without inserting the x1, x2, y1, y2 parameters. This information can be found in the GitHub documentation.

The following text has been added to the Conclusions section in the revised manuscript:
"The pyDHM library is posted publicly on GitHub (65, 66). The GitHub repository includes a complete documentation of the functions implemented, sample codes, and troubleshooting guidelines for correctly using the library. To increase the applicability of this library in our community, the GitHub repository also includes simulated and experimental holograms and some instructional videos on how to install and use the library (67-70)."

Comment #5: "It is understandable that the pyDHM library serves as the first toolbox library that supports multimodality DHM numerical processing. However, its useability is poor at current stage. Immediate future work should be aiming to improve the useability with for example, a graphical user interface. There is no doubt that the spatial filtering process of the +/-1 orders in the FT of an off-axis hologram can be greatly simplified if performed in a graphical way."
Response: We respectfully disagree with the reviewer regarding the useability of the current/revised library since the pyDHM library is not aimed at biology/medicine users. We believe the immediate users of the library are coders with a background in Physics and computational sciences. Nonetheless, we understand the reviewer's point of view and agree that future work should also be focused on implementing a graphical user interface (GUI) for non-coder users. For this reason, the GUI is currently under development by undergraduate students from Physics Engineering and Computational Engineering. We plan to test the usability of the GUI with our biological collaborators.

The following text has been added to the revised manuscript in the Conclusions section:
"Because of the broad applicability of DHM systems in biology and medicine, we will create a graphical user interface (GUI) for the pyDHM library, aiming that users who lack coding skills and background in Optics and DHM could adopt this library."

Regarding the comment about the spatial filtering process from the hologram spectrum implemented in the original library, we agree with the reviewer that some type of graphical interface can facilitate this task. For this reason, we have implemented additional functionalities to simplify the user's usability. For the rectangular and circular spatial filtering functions (*'sfr'* and *'sfc'* defined in the *utility* package, Table 1), we have allow the display of the resulting filtered object spectrum from the hologram's spectrum so that users can verify that all object frequencies have been correctly cropped. On the other hand, a new function for manual spatially filtering has been added to the library (see *sfmr* in Table 1). This function filter the Fourier transform of a hologram using a user-defined rectangular mask. The required parameters are the hologram

(*field*) and the Boolean parameter (*display*) that allows the display of the filtered hologram spectrum when it is true. It is important to mention that this function works only if the OpenCV library is installed. Finally, we have added a new option into the *CNT* function (e.g., the phase compensation function for non-telecentric holograms). This option allows the user to decide whether he/she manually inserts the pixel coordinates for the rectangular mask to spatially filter the object frequencies from the hologram spectrum, or manually selects the object frequencies using a popup window. The latter option is done by calling the function with the parameter *spatialFilter == 'sfmr'*.

The following description has been added in Table 1 of the revised manuscript:

**Circular filter**	sfc(field, radius, centX, centY, display)<br><br>Function to filter the Fourier Transform of a hologram using a circular mask. The required parameters are: *field* the hologram, *radius* is the radius of the circular mask in pixels, and (*centX*, *centY*) are the central pixel positions for the circular mask. The Boolean parameter *display* shows the filtered object frequencies from the hologram spectrum when its value is true.
**Rectangular filter**	sfr(field, x1, x2, y1, y2, display)<br><br>Function to filter the Fourier Transform of a hologram using a rectangular mask. The required parameters are: *field* the hologram; (*x1*, *y1*) the pixel coordinates of the upper left corner for the rectangular mask; and (*x2*, *y2*) the pixel coordinates of the lower right corner for the rectangular mask. The Boolean parameter *display* shows the filtered object frequencies from the hologram spectrum when its value is true.
**Manual rectangular filter**	sfmr(field, display)<br><br>Function to filter the Fourier Transform of a hologram using a user-defined rectangular mask from a popup window. The required parameters are the hologram (*field*) and the Boolean parameter (*display*) that allows the display of the filtered hologram spectrum when it is true. It is important to mention that this function works only if the OpenCV library is installed.

The following description has been added in Table 3 of the revised manuscript:

**Compensation no-telecentric regime**	CNT(inp, wavelength, dx, dy, x1, x2, y1, y2, spatialFilter):<br><br>Function to reconstruct fully-compensated phase images of holograms recorded in non-telecentric regime. The function requires up to nine parameters: *inp, wavelength, dx, dy, x1, x2, y1, y2*. The units of *wavelength, dx, dy* should be microns. The DHM system should operate in off-axis configuration and non-telecentric regime. The parameter *spatialFilter* allows to decide how to select the object frequencies from the hologram spectrum. Use *spatialFilter = 'sfmr'* for a manual filter via a popup windown or *spatialFilter = 'sfr'* for a rectangular mask filter defined by the *x1, x2, y1, y2* parameters. Users selecting the *sfmr* option should not insert any parameter for *x1, x2, y1,* and *y2*. In other words, the notation of the function is CNT(inp, wavelength, dx, dy, x1, x2, y1, y2, spatialFilter ='sfr') or CNT(inp, wavelength, dx, dy, spatialFilter = 'sfmr'). This function is based on the proposed method by Kemper *et al.* (59). We have

	implemented a search algorithm with nested for loops (53) to automatically find the best-reconstructed phase image (e.g. fully-compensate).

Comment #6: "In Fig. 6a, line 7 'hologram' is not defined and an error is prompted when the sample code is run, possibly replacing by 'inp'. Line 9 'ft_holo' should be 'ftholo'. It is recommended for the authors to double check the variable names in sample codes throughout the manuscript."
Response: We thank the reviewer for his/her careful revision. We have corrected these typos and followed his/her recommendation. All the sample codes shown in the manuscript have been double checked. Figures 3 to 8 have been corrected accordingly (see revised text).

Comment #7: "In Fig. 6a, running line 11 – 'output = phaseCompensation.CNT(inp, 0.633, 6.9, 6.9, 200, 287, 180, 267)' in Python gives an error 'TypeError: CNT() missing 3 required positional arguments: 'cur', 's', and 'step'' and prevent further trails. The authors should conduct a thorough proof on the codes before publishing."
Response: The sample code in Fig 6a has been corrected, and now it works perfectly. We have also double-checked all sample codes and all implemented functions. Please refer to comment #6 of Reviewer 1.

Comment #8: "Currently, the pyDHM library only permits single hologram processing, which can be highly inefficient when users have a series of holograms or a video to process. To expand the temporal processing capability of the pyDHM library, the authors should provide a future plan on how this function can be achieved."
Response: We appreciate the reviewer's comment. We agree that an additional functionality should be included to process a sequence/video of holograms for the temporal-dependant applications in DHM. Current plans involve the processing of the hologram sequence through the pyDHM app (which is currently under development). In this app, users can input a single hologram or a sequence/video of holograms, enabling the processing of the whole hologram series. To optimize such video processing, aiming to provide a fast approach, we plan to avoid some reprocessing steps such as the selection of the spatial filter mask. Our final goal is to expand such app further so that pyDHM can be used to analyze biological systems, including motility analysis for microorganism tracking and cell counting. As a final additional functionality, we are currently exploring an automatic algorithm to reconstruct DHM holograms without prior knowledge of the DHM configuration (e.g., only hologram, source's wavelength and sensor's pixel size).

To address the reviewer's suggestion, we have added the following lines to the Conclusions section in the revised manuscript:
"In future works, we will expand the codes within our library and reduce its processing time using GPU implementations. Current implementations within the *pyDHM* library require that the users select the adequate reconstruction method based on the optical configuration of the DHM systems. Future work will focus on an automatic algorithm to reconstruct DHM holograms without prior knowledge of the DHM configuration (e.g., only hologram, source's wavelength, and sensor's pixel size). Because of the broad applicability of DHM systems in biology and medicine, we will create a graphical user interface (GUI) for the pyDHM library, aiming that users who lack coding skills and background in Optics and DHM could adopt this library. Such an app will allow the users to input a single hologram or a sequence/video of holograms, enabling the processing of the whole hologram series. Some reprocessing steps will be avoided, such as selecting the spatial filter mask to optimize such video processing. Our final goal is to expand such app further so that pyDHM can be used to analyze biological systems, including motility analysis for microorganism tracking and cell counting."

Reviewer 2

Comment #0: "The manuscript is written clearly and understandably. An extensive literature review has been provided, and delineation to the literature has been presented. In-depth knowledge of digital holographic microscopy and detailed explanations of the advantages and disadvantages of various techniques and algorithms are provided. The authors contribute to community efforts by sharing the software publicly on Github. Code examples are available, which facilitates the introduction to the package."
Response: We appreciate the reviewer for reading the manuscript and providing positive feedback.

Comment #1: "The software is documented in the manuscript, however I would also suggest to add a documentation on github or dedicated software documentation services."
Response: The GitHub repository has been revised to include complete documentation for the pyDHM library. Please refer to comment 2 of Reviewer 1.

Comment #2: "I believe the manuscript and the python package are useful contributions to the digital holographic microscopy community."
Response: Thank you so much.
